# Differential regulation of osteopontin and CD44 correlates with infertility status in PCOS patients

**DOI:** 10.1007/s00109-020-01985-w

**Published:** 2020-10-13

**Authors:** R. Paravati, N. De Mello, E. K. Onyido, L. W. Francis, K. Brüsehafer, K. Younas, S. Spencer-Harty, R. S. Conlan, D. Gonzalez, Lavinia Margarit

**Affiliations:** 1 Reproductive Biology and Gynaecological Oncology Group, Institute for Life Science 2, School of Medicine, Swansea University, Singleton Park, Swansea, SA2 8PP UK; 2 Swansea Bay University Health Board, Obstetrics Gynaecology Department, Singleton Hospital, Sketty Lane, Swansea, SA2 8QA UK; 3 Swansea Bay University Health Board, Cellular Pathology Department, Singleton Hospital, Sketty Lane, Swansea, SA2 8QA UK; 4 Cwm Taf Morgannwg University Health Board, Obstetrics Gynaecology Department, Princess of Wales Hospital, Coity Road, Bridgend, CF31 1RQ UK

**Keywords:** CD44, OPN, Cytokines, Infertility, Endometrium

## Abstract

**Abstract:**

Endometrial receptivity is mediated by adhesion molecules at the endometrium-trophoblast interface where osteopontin (OPN) and CD44 form a protein complex that plays an important role in embryo recognition. Here, we undertook a prospective study investigating the expression and regulation of OPN and CD44 in 50 fertile and 31 infertile ovulatory polycystic ovarian syndrome (PCOS) patients in the proliferative and secretory phases of the natural menstrual cycle and in 12 infertile anovulatory PCOS patients. Endometrial biopsies and blood samples were evaluated for expression of OPN and CD44 using RT-PCR, immunohistochemistry and ELISA analysis to determine circulating levels of OPN, CD44, TNF-α, IFN-γ and OPN and CD44 levels in biopsy media. Our findings highlighted an increased level of circulating OPN and CD44 in serum from infertile patients that inversely correlated with expression levels in endometrial tissue and positively correlated with levels secreted into biopsy media. OPN and CD44 levels positively correlated to each other in serum and media from fertile and PCOS patients, as well as to circulating TNF-α and IFN-γ. In vitro analysis revealed that hormone treatment induced recruitment of ERα to the OPN and CD44 promoters with a concomitant increase in the expression of these genes. In infertile patients, inflammatory cytokines led to recruitment of NF-κB and STAT1 proteins to the OPN and CD44 promoters, resulting in their overexpression. These observations suggest that the endometrial epithelial OPN-CD44 adhesion complex is deficient in ovulatory PCOS patients and displays an altered stoichiometry in anovulatory patients, which in both cases may perturb apposition. This, together with elevated circulating and local secreted levels of these proteins, may hinder endometrium-trophoblast interactions by saturating OPN and CD44 receptors on the surface of the blastocyst, thereby contributing to the infertility associated with ovulating PCOS patients.

**Key messages:**

• Endometrial epithelial OPN-CD44 adhesion complex levels are deficient in ovulatory PCOS patients contributing to the endometrial infertility associated with ovulating PCOS patients.

• Circulating levels of OPN, CD44 and inflammatory cytokines TNF-α and IFN-γ are altered in infertile PCOS patients.

• Increased levels of both OPN and CD44 in biopsy media and serum inversely correlate with endometrial expression of these markers in endometrial tissue.

• In infertile PCOS patients, high levels of oestrogens and inflammatory cytokines stimulate the recruitment of transcription factors to the OPN and CD44 promoters to enhance gene transcription.

• Our study identifies a novel crosstalk between the CD44-OPN adhesion complex, ERα, STAT1 and NF-κB pathways modulating endometrial receptivity.

**Electronic supplementary material:**

The online version of this article (10.1007/s00109-020-01985-w) contains supplementary material, which is available to authorized users.

## Introduction

Endometrial receptivity depends on synchronous biochemical, structural and molecular events for successful embryo implantation to occur during the window of implantation at day 20 to 24 in the menstrual cycle. This is characterised by optimal hormone levels, cytokine signalling and the presence of luminal endometrial adhesion proteins, including osteopontin (OPN) and CD44, to support the embryo attachment [1, 2].

Infertility conditions, including polycystic ovary syndrome (PCOS), are associated with defective endometrial expression of adhesion proteins [3]. In the fertile endometrium, expression of the oestrogen and progesterone nuclear hormone receptors, ERα and PR, is elevated during the proliferative phase of the menstrual cycle, and then reduced with the rise of progesterone after ovulation. However, in infertile PCOS patients, expression of ERα and PR is elevated, leading to altered expression of ERα- and PR-regulated proteins involved in implantation [4].

CD44 is a transmembrane glycoprotein expressed during the window of implantation [5] and has been implicated in the migration and adhesion of endometrial cells [6]. The phospho-glycoprotein, OPN, is also expressed during the window of implantation acting as a bridging molecule between the endometrial luminal surface and the trophoblast through interactions with αvβ3 integrin and CD44 [5]. In the normal secretory phase, endometrium CD44 expression is increased [7], mirroring the expression of OPN, thus supporting a role for CD44 in facilitating attachment of the blastocyst [8].

Variation of OPN and CD44 expression during the menstrual cycle [9, 10] and the presence of regulatory elements for ERα in the promoter regions of both OPN and CD44 suggest that their expression is regulated by oestrogen, as well as progesterone acting through PR isoform B (PRB), at least for OPN [11]. In the normal endometrium, oestrogen also upregulates the expression of a number of inflammatory cytokines including TNF-α, and IFN-γ, each of which has been implicated in endometrium receptivity, with increased expression at the time of implantation [12, 13]. TNF-α, a potent pro-inflammatory cytokine, is involved in the cyclic endometrial regeneration [14]. IFN-γ induction also plays a key role in implantation, particularly in the vascular remodelling process through activation of the STAT1 pathway [15, 16]. In particular, alterations of the endometrial cytokine pattern have been shown to influence the implantation process, with direct correlation between recurrent miscarriage and high levels of IFN-γ, and TNF-α reported [17]. Several studies have proposed that pro-inflammatory molecules play a role in the complex inflammatory cascade that is associated with PCOS [18]. For instance, elevated levels of TNF-α, TNFR2 receptors and nuclear p65 have been reported in secretory endometrial tissue of PCOS women, suggesting a sustained pro-inflammatory uterine environment through enhanced activation of p65-NF-κB pathway in these patients [19].

Therefore, this study aims to evaluate the endometrial expression and circulating levels of CD44 and OPN proteins in infertile patients diagnosed with PCOS. The effects of steroid hormones and pro-inflammatory cytokines on CD44 and OPN gene and protein expressions were also assessed using in vitro models. We hypothesised that changes in the ratios of endometrial versus serum levels of these two molecules in PCOS patients could lead to alterations in the stoichiometry of the OPN-CD44 adhesion complex at the endometrial surface, thus promoting the saturation of OPN and CD44 receptors on the surface of the blastocyst by secreted molecules. Together, such changes could prevent endometrium-trophoblast interactions contributing to endometrial infertility in PCOS patients.

## Materials and methods

### Patients

The control group consisted of women with proven fertility, all parous (parity 1–5) and with confirmed ovulation in the tested cycles. These patients were recruited from general gynaecology clinics, having presented for sterilisation with no confirmed diagnosis of PCOS. The infertile group consisted of women diagnosed with PCOS. Endometrial biopsies were obtained from women in a natural menstrual cycle whose phase was confirmed by ultrasound, hormonal and histological criteria. A urinary LH test was also used to confirm ovulation, and in post-ovulatory cycles, the sample was taken on day 6–8 post LH surge. All patients were receiving no exogenous hormonal treatment for at least 2 months prior to the procedure. PCOS was defined using the Rotterdam criteria by the presence of two or more features of clinical and biochemical hyperandrogenism, oligo-anovulation and/or polycystic ovaries (ultrasound) [20]. Related disorders such as tumours producing androgens or patients with Cushing’s syndrome were excluded.

Ovulatory PCOS patients had confirmed polycystic ovaries on ultrasound and hyperandrogenism; ovulated spontaneously, with serum progesterone levels measured at LH+7 at least 30 nm/L; and were infertile despite regular ovulatory cycles in the presence of patent tubes and normal sperm parameters. Tubal patency was confirmed by either a HyCoSy scan or laparoscopy and dye test. The patients in control and study groups were matched for body mass index and smoking habits.

Samples for histological evaluation and immunohistochemical studies were taken with a sterile Pipelle endometrial suction for sampling of the functional layer of the endometrium [21]. Blood samples were collected from patients at mid proliferative (days 5 to 8) for routine fertility assessment of the hormonal panel and at secretory phase (day 21, LH+7) for progesterone, CD44, OPN and inflammatory cytokine levels. Biopsies were taken at proliferative and secretory phases timed with the blood sampling. Proliferative phase circulating androgen levels were also assessed in the fertile and PCOS patients. Patients with diagnosed coincident uterine pathology that could modify the endometrial structure and function (as hyperplasia, endometrial polyp or endometritis) were excluded from the study. Ethical approval was obtained from the South Wales Local Research Ethics Committee (Wales 6 reference 05/WMW02/103 and 12/WA/0289); written consent was obtained from all patients at the time of recruitment. The study duration was 2 years from patient recruitment to data collection and analysis.

### Assessment of hormone levels in serum

Electrochemiluminescence immunoassays (ECLIA, Roche) were used to measure the serum levels of testosterone (Elecsys® Testosterone II assay), sex hormone–binding globulin (SHBG, Elecsys® SHBG assay), dehydroepiandrosterone sulfate (DHEAS Elecsys® assay), progesterone (Elecsys® Progesterone II), LH (Elecsys® LH) and FSH (Elecsys® FSH) using an Elecsys 2010 immunoassay analyser (Roche). Androstenedione was assayed using RIA (Beckman Coulter). Samples with total testosterone levels over 1.8 nmol/l were also analysed by LC-MS to confirm values, as per NHS Wales policy; these values are reported in Table 1.

**Table 1:** Patients’ clinical data

	Fertile	Ovulatory PCOS	Anovulatory PCOS
Age	30 ± 4.6	29 ± 5.3(*p* = 0.7002)	29.1 ± 2.55(*p* = 0.562)
BMI (kg/m^2^)	26.78 ± 4.96	29.01 ± 5.02(*p* = 0.520)	32.33 ± 5.81(*p* = 0.076)
Progesterone day 21 (ng/ml)	31 ± 5.28	27.45 ± 11.5(*p* = 0.597)	N/A
FSH (mUI/ml)	7.4 ± 3.017	5.67 ± 1.37(*p* = 0.135)	5.34 ± 2.57(*p* = 0.2041)
LH (mUI/ml)	4.90 ± 2.221	12.70 ± 4.27(*p* = 0.0004)	15.63 ± 4.94(*p* = 0.0008)
FSH/LH ratio	1.623 ± 0.57	0.48 ± 0.151(*p* = 0.0001)	0.363 ± 0.19(*p* = 0.0005)
Total T (nmol/l)	0.76 ± 0.472	1.72 ± 0.313(*p* = 0.1)	2.94 ± 0.108(*p* = 0.001)
Free T (pmol/l)	13.13 ± 6.12	18.5 ± 5.7(*p* = 0.08)	37.15 ± 10.6(*p* = 0.002)
Free androgen index	4.10 ± 0.81	5.6 ± 0.39(*p* = 0.05)	8.12 ± 1.16(*p* = 0.003)
SHBG (nmol/l)	42.60 ± 5.18	34.51 ± 2.97(*p* = 0.8)	27.92 ± 10.5(*p* = 0.05)
A (nmol/l)	3.43 ± 0.23	7.70 ± 1.6(*p* = 0.006)	8.72 ± 1.1(*p* = 0.0001)
DHEAS (μmol/l)	5.07 ± 0.76	7.26 ± 0.61(*p* = 0.03)	7.560. ± 0.32(*p* = 0.01)

Values are mean ± SD*BMI* body mass index, *FSH* follicle-stimulating hormone, *LH* luteinising hormone, *T* testosterone, *A* androstenedione, *DHEAS* dehydroepiandrosterone

### Immunohistochemistry

Samples were fixed in 10% buffered formaldehyde for 24 h and embedded in paraffin wax, and 3–4-μm-thick sections prepared on positively charged slides for immunohistochemical studies. The sections were de-waxed using a Roche dewaxing solution. The tissue sections were incubated with rabbit anti-human OPN polyclonal antibody (AB1870; Millipore) and rabbit anti-CD44 monoclonal antibody (clone EPR1013Y; Millipore), both diluted 1:100. Rabbit IgG was used as negative control, and adjacent sections were cut and stained in parallel using identical procedures. Renal cell cancer and tonsil positive control were used for OPN and CD44 respectively.

For antigen retrieval, the slides were incubated in CC1 buffer (Ventana Biotek Solutions, Tucson, AZ) for an hour on heated plates at 100 °C on a Benchmark XT processor. Primary antibody incubation was for 36 min at dilution 1:100 at 37 °C. Positive immunostaining was detected through interaction of avidin-biotin peroxidase (ABC) complex with biotin conjugated secondary antibody using a Ventana I View DAB detection kit (Ventana Biotek Solutions, Tucson, AZ) and avidin-biotin blocker. The slides were subsequently counterstained with haematoxylin, dehydrated, cleared and mounted in DPX mountant to be examined under light microscopy. We used an immunohistochemical scoring system (IHC) in which the observers perform a thorough examination of all the immunohistochemical sections of the tissue slide using a multi-headed microscope [22]. The endometrial epithelium was assessed separately for the lumen and glands and scored for intensity and distribution of staining. The intensity of staining was scored from (0)—absent to (4)—strong. The distribution of staining was assessed as follows: (0)—absent, (1)—less than 30%, (2)—30 to 60%, (3)—more than 60% and (4)—100% of the tissue surface stained (H-score). The observers were blinded to the patients’ diagnosis, demographics and timing in the cycle of endometrial biopsy.

### Cell culture

Regulation of OPN and CD44 was assessed in the Ishikawa endometrial epithelial cell line (ECACC 99040201, STR authentication, Public Health England, UK), a well-differentiated human endometrial adenocarcinoma cell line, expressing both ERα and PR A & B receptors, regulated in a manner similar to that of normal endometrium [23]. Ishikawa cell lines at low passage number (passage number ≤ 22) were cultured at 37 °C with 5% CO_2_; at least 24 h prior to experiments, the medium was changed to phenol red-free media with 10% charcoal stripped FCS. Cells were stimulated with pro-inflammatory cytokines TNF-α (25 ng/ml, 4 h) (Miltenyi Biotec UK, Cat no. 130-094-014) and IFN-γ (2 IU, 24 h) (Miltenyi Biotec UK, Cat no. 130-096-872) separately, or steroid hormones 17-β-oestradiol (E_2_; 10 nM, 48 h) (Sigma-Aldrich, UK Cat-No E8875) and progesterone (P_4_; 1 μM, 48 h) (Sigma-Aldrich UK, Cat-No M1629) alone or in combination as previously described [4, 24]. Following stimulation, cell pellets were collected for mRNA and chromatin immunoprecipitation analysis. The presence of ERα, PRA and PRB receptors in Ishikawa cells was confirmed by immunoblots (data not shown).

### Immunoblot

Proteins were quantified (Bradford assay) and equal amounts (20 μg) resolved by SDS-PAGE, transferred to PVDF membranes and blocked overnight with 10% BSA, in 0.1% Tween-20-TBS (TTBS). Membranes were subsequently incubated at 4 °C with rabbit anti-human ERα rabbit polyclonal antibody (HC-20 Santa Cruz Biotechnology, USA) (diluted 1/500 in 5% BSA-TTBS buffer) or anti-human PR A/B rabbit polyclonal antibody (H-190 Santa Cruz Biotechnology, USA) (diluted 1/1000 in 5% BSA-TTBS buffer). Blots were then incubated for 1 h with IgG horseradish peroxidase secondary antibody diluted 1/2000 in 5% BSA-TTBS buffer. Between incubation steps, membranes were washed several times with TTBS. Blots were analysed for GAPDH levels (GAPDH rabbit polyclonal antibody (FL-335, Santa Cruz Biotechnology, USA)) to normalise protein loading in each well. Immunoreactive bands were visualised using a ChemiDoc System Bio-Rad Imager (Bio-Rad) and quantified by Quantity One® Imaging software (Bio-Rad), as described previously [3].

### RNA isolation and qPCR

Endometrial biopsies were snap-frozen in liquid nitrogen immediately following excision during the surgical procedure and further stored in liquid nitrogen until subsequent analyses. Total RNA was isolated from snap frozen biopsies and cell line using a RNeasy mini Kit (Qiagen, UK). DNase-I-treated RNA was reverse transcribed into cDNA (high-capacity cDNA conversion; Applied Biosciences, UK) before assessing CD44 and OPN expression using specific primer pairs available on request (Beacon Design 2.0; Premier Biosoft International, USA). qPCR amplification was performed in triplicate in 96-well plates in a Bio-Rad IQ iCycler. Serial dilutions of cDNA were used to plot a calibration curve, and gene expression was quantified by plotting threshold cycle values. Expression levels were normalised to values obtained for the reference gene Ribosomal Protein 60S L 19 (RPL-19) [4].

### Enzyme-linked immunosorbent assay

Serum samples (50 μl) were collected following centrifugation of blood samples at 1500 g for 10 min and stored at −20 °C. Biopsy media was collected, centrifuged and stored as described above prior to enzyme-linked immunosorbent assay (ELISA) tests. The levels of CD44 (CD44 Human ELISA Kit, Abcam, UK), OPN (Human OPN DuoSet ELISA R&D Systems, UK), TNF-α (Human TNF-alpha DuoSet ELISA, R&D Systems, UK) and IFN-γ (Human IFN-gamma DuoSet ELISA, R&D Systems, UK) were measured in serum and in biopsy media by ELISA following the manufacturer’s protocol. Samples were analysed in triplicate and data obtained for in vitro experiments is representative of four independent experiments.

### Chromatin immunoprecipitation analysis

Ishikawa cell sample fixation, DNA shearing and chromatin immunoprecipitation (ChIP) were performed following the manufacturer’s instructions (Porvair Sciences, UK). Chromatin was quantified using a Nanodrop spectrophotometer and visualised using agarose gel electrophoresis to ensure correct distribution of fragment sizes prior to immunoprecipitation. Four hundred nanograms of each chromatin sample was used per ChIP with 0.8 μg of the relevant antibody; NF-κB anti-p65 (C-20, Santa Cruz Biotechnology, USA), STAT1 (phospho Y701, ab30645, AbCam, UK), anti-ERα (HC-20, Santa Cruz Biotechnology, USA) and non-specific rabbit IgG (Rockland Inc., USA) antibodies were used. Enriched fragments were reverse cross-linked, and protein removed by protease digestion prior to qPCR. Specific primer sets (available on request) for predicted NF-κB, STAT1 and ERα binding sites in the OPN and CD44 promoters were used to assess promoter occupancy by qPCR, which was performed as described [25].

### Statistical analysis

Data distributions were assessed for normality using the Kolmogorov-Smirnov tests. Non-normally distributed data were analysed with the Mann-Whitney *U* test applied post hoc to determine statistical significance. For normally distributed data, an ANOVA test followed by a *t* test was used to determine significant differences between groups. The test statistic and corresponding *P* value were reported. Correlations between CD44 and OPN immunostaining and OPN and CD44 serum levels in each group were assessed by the nonparametric Spearman test. Correlations between TNF and IFN serum levels with OPN and CD44 levels in media and serum were performed via Pearson correlation coefficient. All data analysis was performed using SPSS version 16.0 (SPSS, Chicago, IL).

## Results

### Clinical data

Ninety-three patients were enrolled in this study: 50 fertile (20—proliferative phase, 30—secretory phase), 31 infertile ovPCOS patients (10—ovulatory in proliferative phase, 21—secretory phase (ovPCOS)) and 12 infertile anovulatory PCOS patients (anovPCOS in proliferative phase). Our previously published observations were used to determine the sample size of the study [4]. There was no statistically significant difference in the mean age and body mass index (BMI) between the fertile and study groups (see Table 1).

The levels of FSH and progesterone were not significantly different between ovulatory groups. LH was significantly higher and an FSH:LH ratio of < 1 was detected in both PCOS groups compared to controls. The PCOS groups expressed significantly higher levels of androstenedione and dehydroepiandtrosterone sulfate (DHEAS). Free testosterone levels were not different from the controls in the ovPCOS patients, but were significantly higher in the anovPCOS group (see Table 1). The SHBG levels were only marginally higher in the anovPCOS group.

### Immunohistochemical evaluation of CD44 and OPN expression in fertile and infertile endometrium

**Fig. 1:**
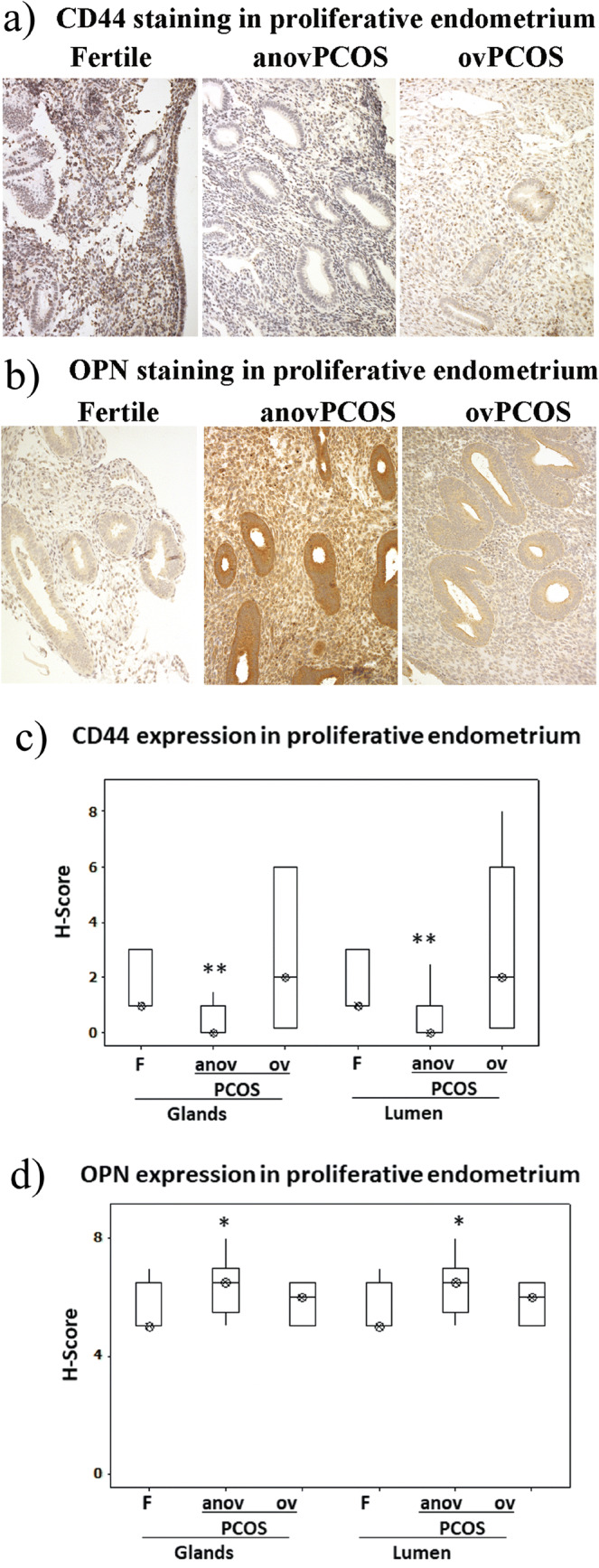
Immunohistochemical assessment of CD44 and OPN expression in proliferative endometrial epithelium. **a** CD44 staining levels in endometrial biopsies showing reduced intensity of CD44 staining in anovPCOS samples compared to fertiles. **b** OPN staining levels in endometrial proliferative biopsies indicate a reduced intensity of OPN staining in anovPCOS endometrium compared to fertile endometrium. Immunohistochemistry staining scores for CD44 and OPN assessed in glands and lumen are shown (panels **c** and **d**). Values are expressed as median (*circled x*) and interquartile range (*box and whisker*)*.* The statistical analysis was performed using Kruskal-Wallis test followed by a Mann-Whitney test. *p ≤ 0.05 and **p ≤ 0.001 are considered significant. F, fertile patients (*n* = 20); anovulatory PCOS (anov PCOS*, n* = 12); ovulatory PCOS (ovPCOS, *n* = 10)

**Fig. 2:**
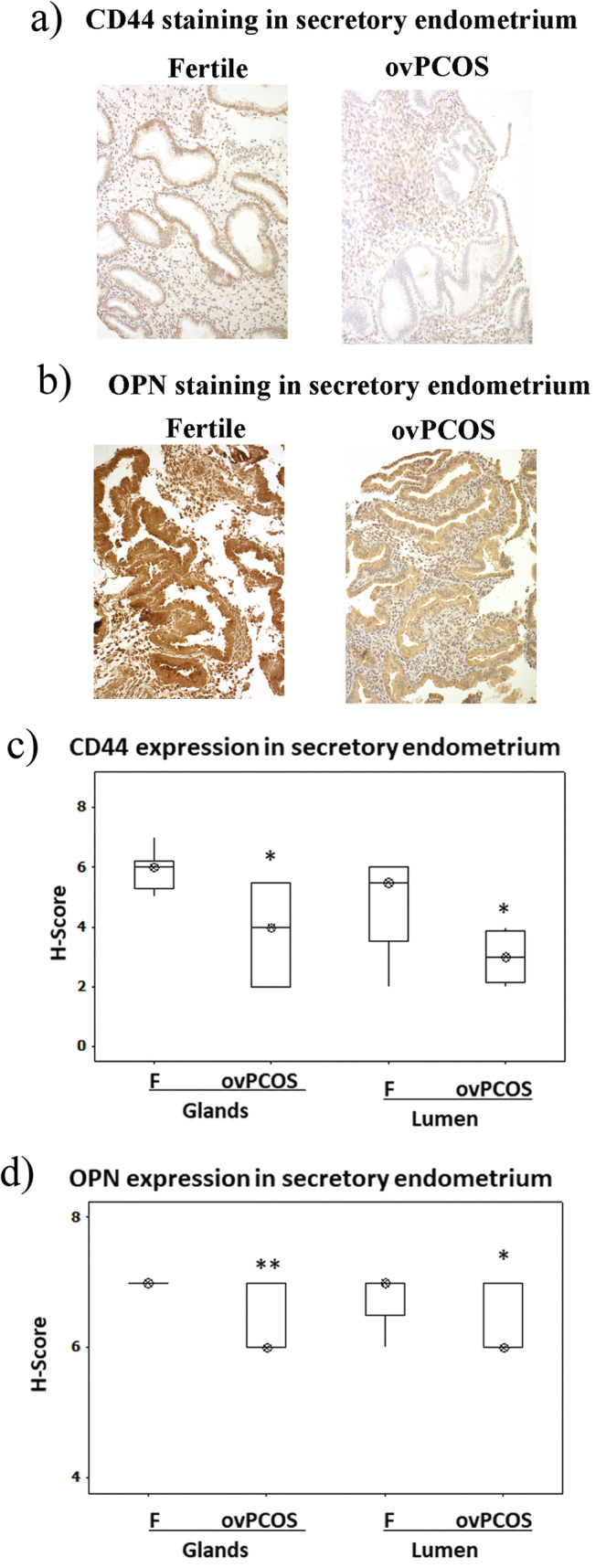
Immunohistochemical assessment of expression of CD44 and OPN in secretory endometrium. **a** The intensity of CD44 staining is significantly reduced in ovulatory PCOS endometrium compared to that of fertile endometrium. **b** OPN staining levels are significantly reduced in ovulatory PCOS compared to those in controls. Immunohistochemistry staining scores for CD44 and OPN assessed in glands and lumen are shown (panels **c** and **d**). Values are expressed as median (*circled x*) and interquartile range (*box and whisker*)*.* The statistical analysis was performed using Kruskal-Wallis test followed by a Mann-Whitney test. *p ≤ 0.05 and **p ≤ 0.001 are considered significant. F, fertile patients (*n* = 30); ovPCOS (*n* = 21)

Expression of CD44 and OPN was assessed in both glandular and luminal endometrial epithelium (see Figs. 1 and 2). In the proliferative phase, endometrium of anovPCOS patients exhibited significantly reduced glandular and luminal staining levels of CD44 (*p* = 0.005, *p* = 0.001) and increased expression of OPN (*p* = 0.045, p = 0.045) compared to fertile endometrium (see Fig. 1). No significant differences were observed in CD44 or OPN expression between the proliferative endometrium of ovPCOS and fertile patients (see Fig. 1).

Differences in OPN and CD44 expression were observed in endometrial glands and lumen of fertile and infertile ovPCOS endometrium during the secretory phase of the menstrual cycle (see Fig. 2). A reduced expression of CD44 staining was observed in secretory phase endometrium of ovPCOS (glands *p* = 0.027, lumen *p* = 0.022) compared to fertile patients (see Fig. 2a, b). Similarly, OPN glandular and luminal staining was reduced in ovPCOS secretory endometrium (glands *p* = 0.001, lumen *p* = 0.05) compared to fertile secretory endometrium (see Fig. 2b, d).

### Circulating levels of OPN, CD44 and inflammatory cytokines TNF-α and IFN-γ are altered in infertile patients

**Fig. 3:**
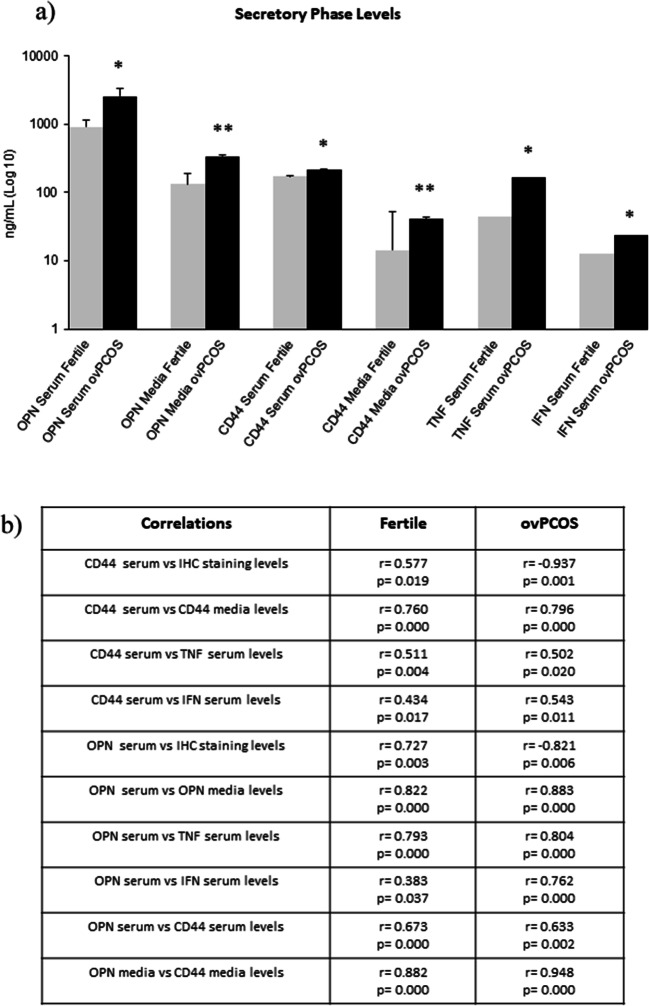
Expression levels of CD44, OPN, TNF-α and IFN-γ in serum and media samples from fertile (*n* = 30) and ovPCOS (*n* = 21) patients measured by ELISA. **a** Expression levels of CD44 (serum and media), OPN (serum and media), serum TNF and serum IFN-γ are significantly higher in ovPCOS patients compared to those in fertile patients during the secretory phase. Values are expressed as log (average) ± SD. Statistical analysis of the data was performed using a Student *t* test. *p ≤ 0.05 and **p ≤ 0.01 are considered significant. **b** Association between expression levels was measured using the Spearman rho coefficients (*r*) for comparisons between IHC scores and serum levels (ELISA), whereas all other associations were analysed using the Pearson correlation test. *p ≤ 0.05 and **p ≤ 0.001 are considered significant. F, fertile patients (*n* = 30); ovPCOS (*n* = 21)

The expression of CD44 and OPN (serum and biopsy media), together with levels of the pro-inflammatory cytokines TNF-α and IFN-γ in serum samples during secretory phase from the patient cohort used for IHC studies, was assessed by ELISA (see Fig. 3). CD44 levels in serum and biopsy media were significantly higher in ovPCOS patients (serum *p* = 0.012; media *p* = 0.000) compared to fertiles indicating that the elevated serum levels originate from endometrial tissue (see Fig. 3a). TNF-α and IFN-γ serum levels were significantly higher in serum from ovPCOS patients (*p* = 0.0214, *p* = 0.0136) compared to fertile patients (see Fig. 3a). Similarly, OPN levels in serum and biopsy media were significantly higher in ovPCOS (serum *p* = 0.001; media *p* = 0.000) patients compared to fertile patients (see Fig. 3a).

Serum levels of CD44 and OPN were positively associated with CD44 and OPN immunostaining levels in endometrium of fertile patients during secretory phase (see Fig. 3b). In contrast, a significant negative association between serum and tissue levels was observed in the ovPCOS group for both OPN and CD44, with high serum and low tissue levels during secretory phase (see Fig. 3b). A significant positive correlation was observed between CD44 and OPN serum levels and the levels of these proteins detected in biopsy media for both fertile and PCOS groups (see Fig. 3b, Fig. 1S and 2S). Additionally, levels of CD44 measured in both serum and media were found to be positively correlated to OPN levels (see Fig. 3b, Fig. 3S). Both OPN and CD44 serum levels were found to be positively correlated to circulating levels of TNF-α and IFN-γ in both fertile and PCOS endometrium (see Fig. 3b, Fig. 1S and 2S). These results support the concept of CD44 and OPN as a functional complex present on endometrial cells, the composition of which alters in PCOS patients, possibly due to alterations in steroid hormones, their receptors and pro-inflammatory cytokines. To understand the mechanisms underlying these changes in CD44 and OPN, in vitro assays were undertaken to determine whether they were due to changes in gene regulation.

### Steroid hormone regulation of CD44 and OPN in vitro

**Fig. 4:**
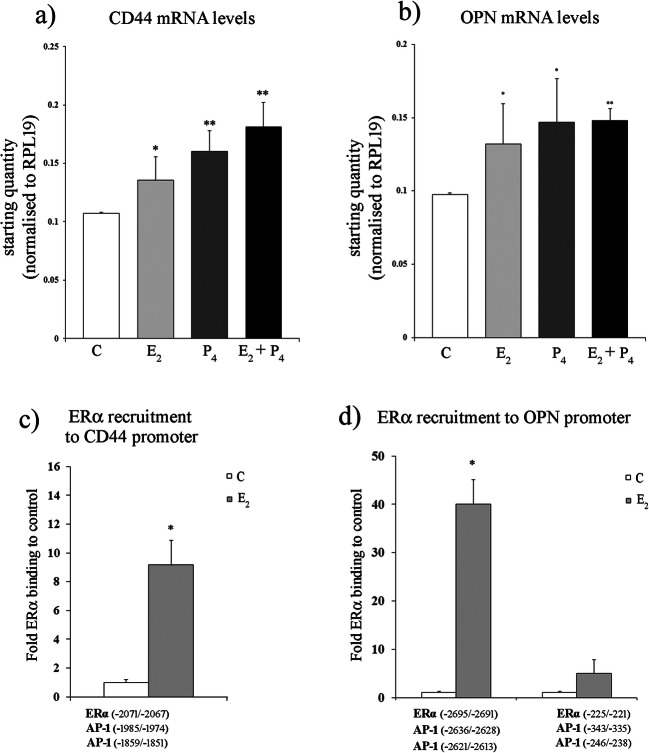
Regulation of OPN and CD44 endometrial expression by oestrogen and progesterone hormones. **a**, **b** Transcript levels of CD44 and OPN in Ishikawa cells treated for 48 h with hormones E_2_ (10 nM) and P_4_ (1 μM) were analysed by qPCR. Values, normalised to RPL19, are expressed as the average ± SD. Results are representative of three biological repeats. Statistical analysis of the data was performed using a Student *t* test. *p ≤ 0.05, **p ≤ 0.01 and ***p ≤ 0.001 are considered significant. **c**, **d** Quantitative analysis of ERα occupancy of CD44 and OPN promoter was assessed after E2 (10 nM) stimulation of Ishikawa cells (ERα +ve) by chromatin immunoprecipitation. Results are representative of three biological repeats. Values are expressed as the average ± SD. Statistical analysis of the data was performed using a Student *t* test. *p ≤ 0.05, **p ≤ 0.01 and ***p ≤ 0.001 are considered significant

The expression of both OPN and CD44 is increased in the secretory phase endometrium of fertile women, suggesting they are regulated by oestrogen and progesterone hormones. Ishikawa is an endometrial epithelial cell line commonly used as an in vitro model for uterine receptivity studies, having functional steroid hormone receptors [26] and expressing CD44 and OPN [27]. Following treatment with E_2_ (*p* = 0.019), P_4_ (*p* = 0.001) or a combination of E_2_ + P_4_, a significant increase in CD44 mRNA was observed in Ishikawa cells (*p* = 0.001) (see Fig. 4a). A similar increase in OPN expression was observed after 48 h treatment (E_2_, *p* = 0.048; P_4_, *p* = 0.020; E_2_ + P_4_, *p* = 0.001) (see Fig. 4b) demonstrating that both CD44 and OPN are positively regulated by E_2_ and P_4_.

The response observed following E_2_ treatment could be due to a direct effect of activated ERα, which can exert its function through targeting oestrogen response elements (EREs), or indirectly via interactions with other transcription factors including AP1 and SP1 [28]. Promoter analysis of OPN and CD44 identified several adjacent ERE, AP1 and SP1 binding sites in both genes; therefore, ERα occupancy was analysed by ChIP (see Fig. 4c, d). A very high and statistically significant increase in ERα occupancy was observed at the EREs in both CD44 (ERE (−2071/−2067), *p* = 0.020) and OPN (ERE (−2695/−2691), *p* = 0.017) promoters 48 h following E_2_ stimulation, thereby confirming that OPN and CD44 are directly regulated by ERα in human endometrial cells. Based on this observation and the increased levels of ERα expression reported in endometrium of PCOS patients [4, 29], it is likely that the increased levels of both OPN and CD44 in endometrial biopsy media and serum are a direct result of an increased occupancy of ERα on the promoter of these genes.

### Expression of CD44 and OPN is stimulated by inflammatory cytokines, and results from increased STAT1 and P65-NF-κB promoter occupancy

**Fig. 5:**
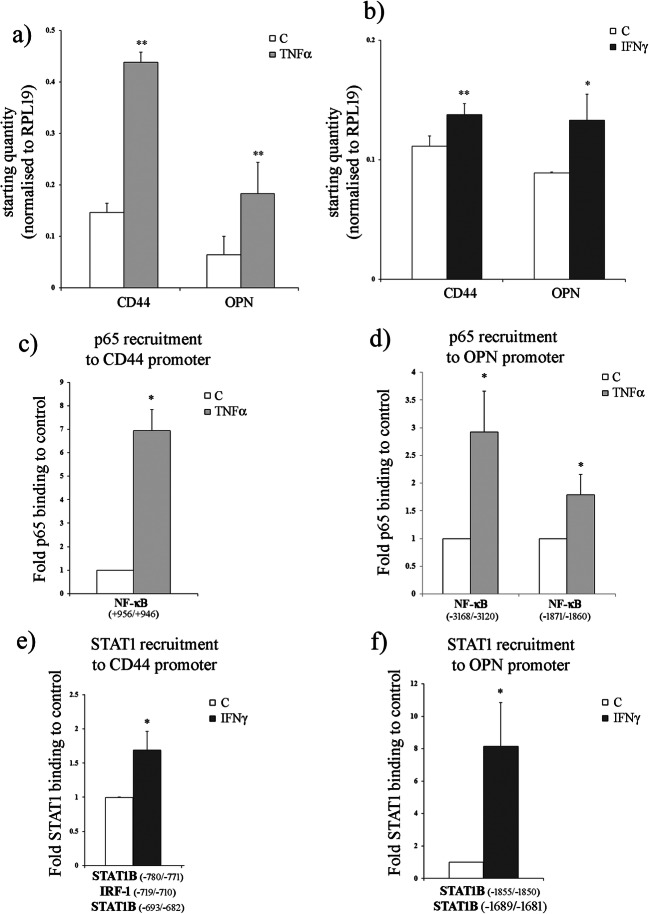
Regulation of OPN and CD44 endometrial expression by TNF-α and IFN-γ. **a**, **b** Transcript levels of OPN and CD44 were analysed by qPCR for Ishikawa cells after treatment with pro-inflammatory cytokines TNF-α (25 ng/ml) or IFN-γ (2 IU). Values, normalised to RPL19, are expressed as the average ± SD. Results are representative of three biological repeats. Statistical analysis of the data was performed using a Student *t* test. *p ≤ 0.05, **p ≤ 0.01 and ***p ≤ 0.001 are considered significant. **c**, **d** Quantitative analysis of CD44 and OPN promoters was conducted using qPCR after TNF-α (25 ng/ml) treatment to measure NF-κB (p65) occupancy in Ishikawa cells. A similar analysis was performed for the CD44 and OPN promoters to measure the occupancy of STAT1 transcription factor after treatment with IFN-γ1b (2 IU) (**e**, **f**). Values are expressed as the average ± SD. Results are representative of three biological repeats. Statistical analysis of the data was performed using a Student *t* test and significance differences described as *p ≤ 0.05, **p ≤ 0.01 and ***p ≤ 0.001

The effects of pro-inflammatory cytokines TNF-α and IFN-γ on CD44 and OPN gene expression were also investigated (see Fig. 5). A significant increase in CD44 (*p* = 0.001) and OPN (*p* = 0.009) expression was observed following treatment with TNF-α after 4 h (see Fig. 5a), and with IFN-γ after 24 h (see Fig. 5b), which also induced CD44 (*p* = 0.001) and OPN (*p* = 0.045) expression.

Activation of NF-κB by TNF-α elicits an inflammatory response, resulting in recruitment of NF-κB to cognate binding sites in many TNF-α regulated genes [30]. Analysis of both CD44 and OPN promoters revealed putative NF-κB binding sites, which we interrogated for occupancy by the NF-κB subunit p65 (see Fig. 5c, d). Increased recruitment of p65 to the CD44 (+956/+946, *p* = 0.03, Fig. 5c) and OPN (−3168/−3120, *p* = 0.01; and 1871/−1860, *p* = 0.02, Fig. 5d) regulatory sites was observed following 4 h TNF-α stimulation. We also analysed whether OPN and CD44 regulation by IFN-γ was mediated by the JAK-STAT pathway, as STAT1 binding sites were identified in the promoters of both genes (see Fig. 5e, f). Significant increases in STAT1 recruitment to STAT1B binding sites in the CD44 (−780/−682, *p* = 0.041; Fig. 5e) and OPN (−1855/−1681, *p* = 0.0306; Fig. 5f) promoters were observed following 24 h of IFN-γ stimulation.

These results demonstrate that CD44 and OPN regulation by inflammatory cytokines TNF-α and IFN-γ is mediated by the recruitment of NF-κB and STAT1 transcription factors to the promoters of these genes, and is likely to occur in the endometrial tissue of PCOS patients where the levels of these two cytokines increase.

## Discussion

OPN and CD44 are adhesion molecules involved in the trophoblast-endometrial interaction, facilitating apposition of the embryo [8, 31]. As well as being expressed in the endometrium, both molecules are secreted by endometrial cells, where they could bind to receptors on the blastocyst blocking interaction with the endometrial surface.

The dysfunctional endometrium of the two PCOS groups is characterised by a similar oestrogenic environment, overexpression of androgen receptor and either diminished (ovPCOS) or lack of (anovPCOS) progesterone activity [32, 33]. We also revealed distinct expression patterns of OPN and CD44 expression in ovulatory and anovulatory PCOS groups during the proliferative phase. In anovPCOS endometrium, which displays only proliferative features, CD44 levels were lower, whereas OPN levels were higher, compared to those of the fertiles. The high levels of OPN in anovPCOS patients could be associated with the levels of oestrogen and androgen, characterising this group of patients as OPN expression is reduced in endometrium cells exposed to the AR inhibitor flutamide and endometrial PCOS cells exhibit an increased expression of ERα nuclear receptor co-activators that may enhance the oestrogen activity in these cells [34, 35]. The decreased levels of CD44 in anovPCOS during proliferative phase mirror the levels observed for ovPCOS in secretory phase promoting the concept of dysfunctional endometrium in PCOS patients, regardless of progesterone action.

Here, decreased levels of OPN and CD44 were observed in the secretory endometrium of ovPCOS patients compared to fertiles, whereas levels of CD44, OPN and pro-inflammatory cytokines were increased in serum, as well as in the media used to collect endometrial biopsies suggesting that circulating CD44 and OPN are of endometrial origin in these infertile patients. In fertiles, serum and endometrial levels of CD44 and OPN showed a positive association, which was also true between CD44 and OPN levels supporting the notion that these proteins form a functional complex on a receptive endometrial cell surface. The increased levels of circulating CD44 and OPN in PCOS serum samples correlate positively with the levels measured in the media in which PCOS biopsies were collected and inversely with endometrial expression levels of these two proteins. These results suggest that the presence of OPN at the endometrial surface is dependent on the expression of its receptor CD44 at the endometrial epithelial cell membrane. Interestingly, these findings agree with studies that reveal that interaction between CD44 and OPN represents a crucial ligand-receptor pair that facilitates systemic insulin resistance [36, 37].

Both OPN and CD44 were directly regulated by ERα occupancy of the cognate ERE promoter site in both genes. In PCOS patients, this nuclear receptor activation is probably due to the local hyperoestrogenic uterine environment in infertile ovulatory PCOS patients and the unopposed oestrogen environment in anovulatory PCOS endometria where oestrogen levels are modulated by hydroxysteroid dehydrogenase (HSD) enzymes [38]. In support of this notion, we have previously described that endometrial levels of 17β HSD-1 are significantly higher in PCOS endometrium compared to those in fertiles [4]. In PCOS patients, inflammatory cytokines are present in the pelvic peritoneal fluid, creating a pro-inflammatory environment in the uterus, which also responds to elevated levels of TNF-α and IFN-γ in the blood of these patients. The elevated expression levels of TNF-α may also facilitate the increase of testosterone levels in PCOS patients [39]. Based on our observations, it appears that these cytokines could directly regulate CD44 and OPN expression through both STAT1 and NF-κB pathways in the endometrium of PCOS women. Given the direct regulation of CD44 and OPN expression by ERα, NF-κB and STAT1, therapeutic interventions that directly target these transcription factors could be beneficial in restoring correct OPN and CD44 expression.

**Fig. 6:**
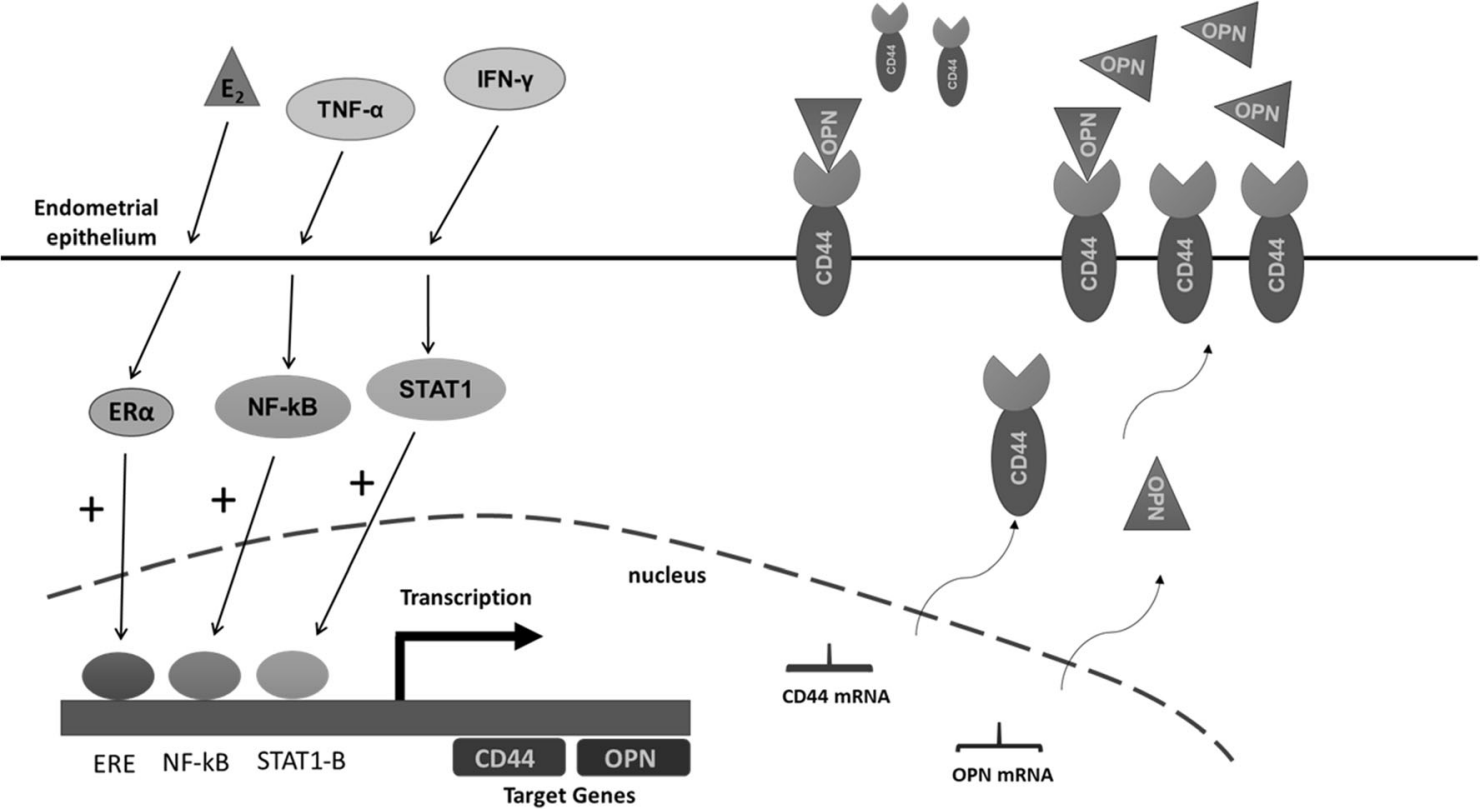
Oestrogen and pro-inflammatory cytokine modulation of CD44 and OPN expression in endometrial epithelial cells. Stimulation of endometrial epithelial cells with oestrogens, TNF-α and IFN-γ leads to activation and translocation of ER, NF-κB and STAT1 proteins to CD44 and OPN promoter to regulate transcription, increasing CD44 and OPN mRNA expression. This upregulation leads to increased CD44 and OPN protein levels at both the secreted and the cell membrane pools. In infertile pathologies, characterised by increased oestrogenic environment with concomitant increased pro-inflammatory cytokine levels, the secreted pool of CD44 and OPN proteins is higher compared to the cell anchored CD44/OPN complex pool. This uterine microenvironment is conducive to abnormal endometrial expression of CD44 and OPN, which could result in sub-optimal implantation

The increased oestrogenic environment, coupled with concomitant increased levels of pro-inflammatory cytokines, appears to create a microenvironment conducive to abnormal endometrial expression of CD44 and OPN (see Fig. 6). This would lead to the establishment of two processes that could contribute to the associated infertility. First, elevated levels of OPN and CD44 circulating molecules, possibly due to increased proteolytic cleavage of CD44, could block these l-selectin receptors on the surface of blastocysts, preventing their attachment to the endometrial surface. This effect would be exacerbated by the abnormal ratio of OPN and CD44 proteins resulting in the formation of non-functional adhesion complexes (anovPCOS) or a simple lack of adhesion complex density on the endometrial epithelium of ovPCOS patients.

Considering the levels of cytokines, CD44 and OPN at tissue and blood levels, it is important to take into consideration in vivo, the dialogue between these markers, the hyperoestrogenic environment and the other non-ovulation-related factors affecting fertilisation and implantation. Notably, the parallel between the tissue and blood values, reported in this study and timed in the menstrual cycle, makes blood biomarkers an attractive less invasive option to implement as part of fertility investigations. It is also important to acknowledge the differences in fertility profiles in women with ovulatory and anovulatory PCOS. Overall, the ovulation and fertilisation processes are just as important as the implantation process, which is dependent on a regular menstrual cycle. Abnormal function at this level can explain the higher incidence of silent and recurrent miscarriages in PCOS women with ovulatory and non-ovulatory cycles.

In conclusion, our findings reveal a novel molecular mechanism which highlights the crosstalk between the CD44-OPN adhesion complex and STAT1 and NF-κB pathways modulating endometrial receptivity. Abnormal CD44-OPN adhesion complex formation could alter trophoblast-endometrial interactions, thereby hindering interactions between the endometrial epithelium and the outer trophectoderm cells of the blastocyst. Therefore, this abnormal circulation of CD44 and OPN can be exploited as a possible biomarker for early diagnosis of infertility and further enhance current clinical practice in the diagnosis of endometrium receptivity status of patients. The identification of treatments to modify the interaction between the ligand/receptor pair of CD44 and OPN may potentially contribute to the improved diagnostic status of infertile PCOS patients.

### Limitations

Limitations include the fact that recruitment of patients for endometrial studies is known to be challenging, and for this reason, the number of endometrial tissue samples remains limited specifically for ovPCOS patients in proliferative phase resulting in the wide interquartile range described in Fig. 1. We are also aware of the difficulty of comparing in vitro results with in vivo findings. Hence, even though we tested steroid hormone and pro-inflammatory cytokine effects in an in vitro environment, we could not consider, in our in vitro model, the roles of systemic hyperoestrogenism or chronic inflammation, both common features in PCOS. Another limitation of the study is that the homeostasis model assessment of insulin resistance (HOMA-IR) [40] is not routinely investigated in clinical practice. Hence, the positive correlation of CD44 and OPN with the altered HOMA was not assessed, but we can confirm that none of the patients recruited in this study had known type 2 diabetes.

## Electronic supplementary material

Figure Supplementary 1 . Significant positive associations between CD44 levels in serum vs CD44 media levels (Panel A & B), serum TNF levels (Panel C & D) and serum IFNγ levels (Panel E & F) in fertile and PCOS patients. Values are expressed as the average ± SD. Statistical analysis of the data was performed using a Student t test and significance differences described as *, *P* ≤ 0.05, **, *P* ≤ 0.01 and ***, *P* ≤ 0.001. Pearson coefficient = r. (PNG 6596 kb)

High Resolution Image (TIF 2703 kb)

Figure Supplementary 2 . Significant positive associations between OPN levels in serum vs OPN media levels (Panel A & B), serum TNF levels (Panel C & D) and serum IFNγ levels (Panel E & F) in fertile and PCOS patients. Values are expressed as the average ± SD. Statistical analysis of the data was performed using a Student t test and significance differences described as *, P ≤ 0.05, **, P ≤ 0.01 and ***, P ≤ 0.001. Pearson coefficient = r. (PNG 6596 kb)

High Resolution Image (TIF 2703 kb)

Figure Supplementary 3 . Positive correlations between serum CD44 and OPN levels in fertiles (A) and PCOS groups (B). Positive correlations between CD44 vs OPN levels in media from fertile (C) and PCOS (D) patients. Values are expressed as the average ± SD. Statistical analysis of the data was performed using a Student t test and significance differences described as *, P ≤ 0.05, **, P ≤ 0.01 and ***, P ≤ 0.001. Pearson coefficient = r. (PNG 6596 kb)

High Resolution Image (TIF 2702 kb)
