# Supplementary figures and images for: Differential regulation of osteopontin and CD44 correlates with infertility status in PCOS patients
Source: J Mol Med (Berl). 2020 Oct 13;98(12):1713–25. doi: 10.1007/s00109-020-01985-w (PMC7679339; doi:10.1007/s00109-020-01985-w)

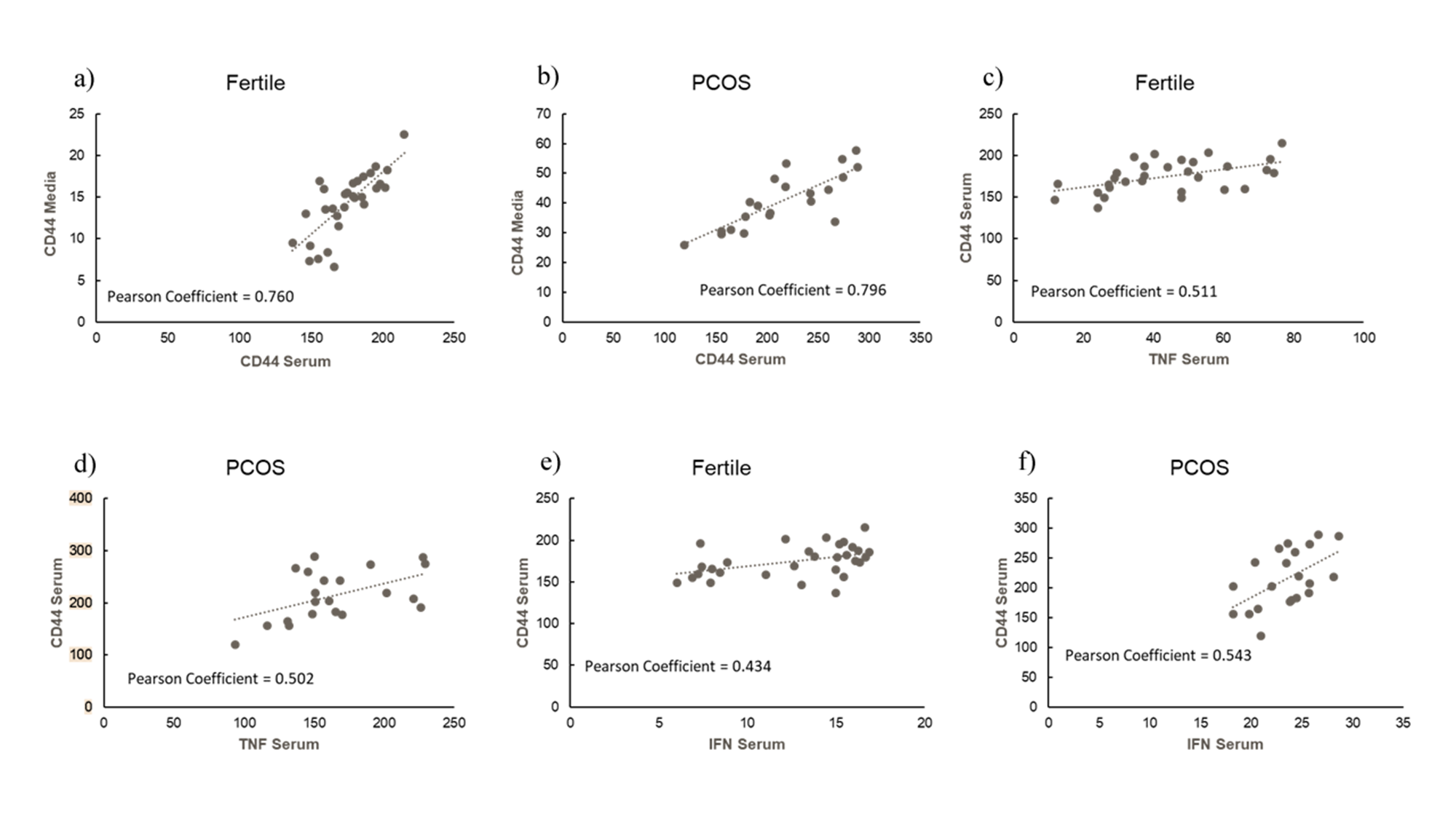

Supplement: Supplementary file 1 — . Significant positive associations between CD44 levels in serum vs CD44 media levels (Panel A & B), serum TNF levels (Panel C & D) and serum IFNγ levels (Panel E & F) in fertile and PCOS patients. Values are expressed as the average ± SD. Statistical analysis of the data was performed using a Student t test and significance differences described as *, P ≤ 0.05, **, P ≤ 0.01 and ***, P ≤ 0.001. Pearson coefficient = r. (PNG 6596 kb) [file 109_2020_1985_Fig7_ESM.png]

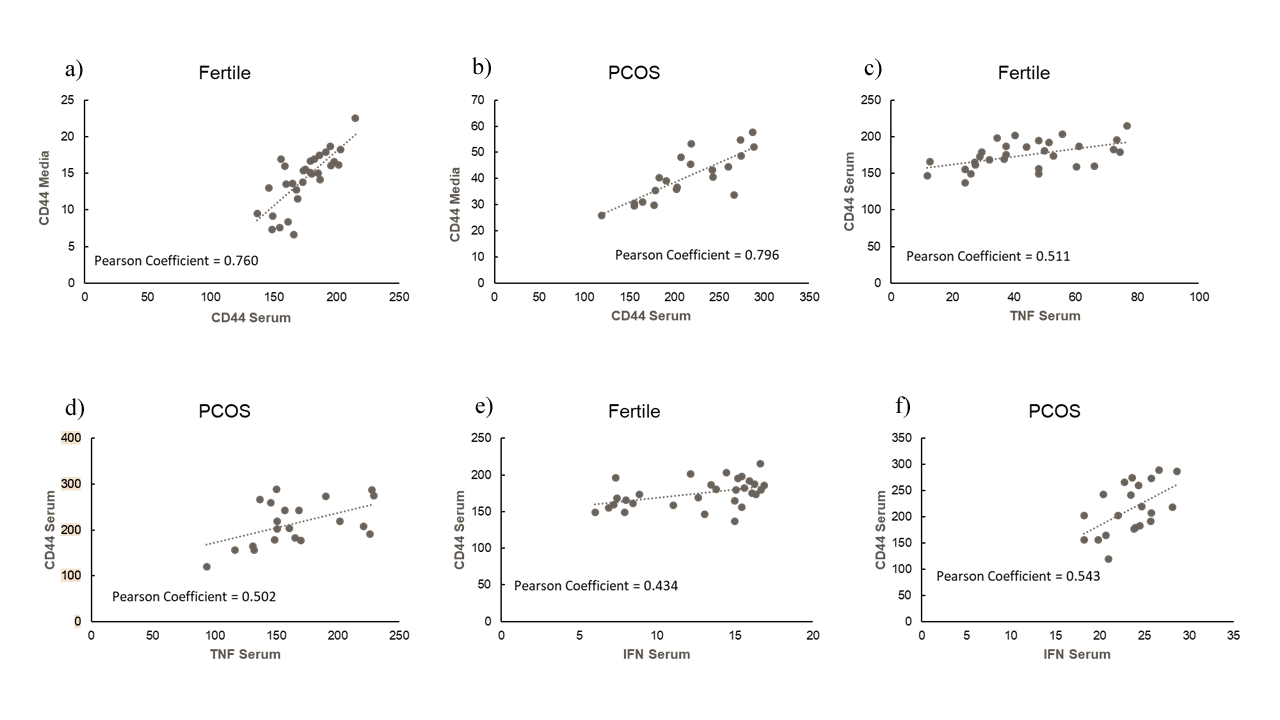

Supplement: Supplementary file 2 — High Resolution Image (TIF 2703 kb) [file 109_2020_1985_MOESM1_ESM.tif]

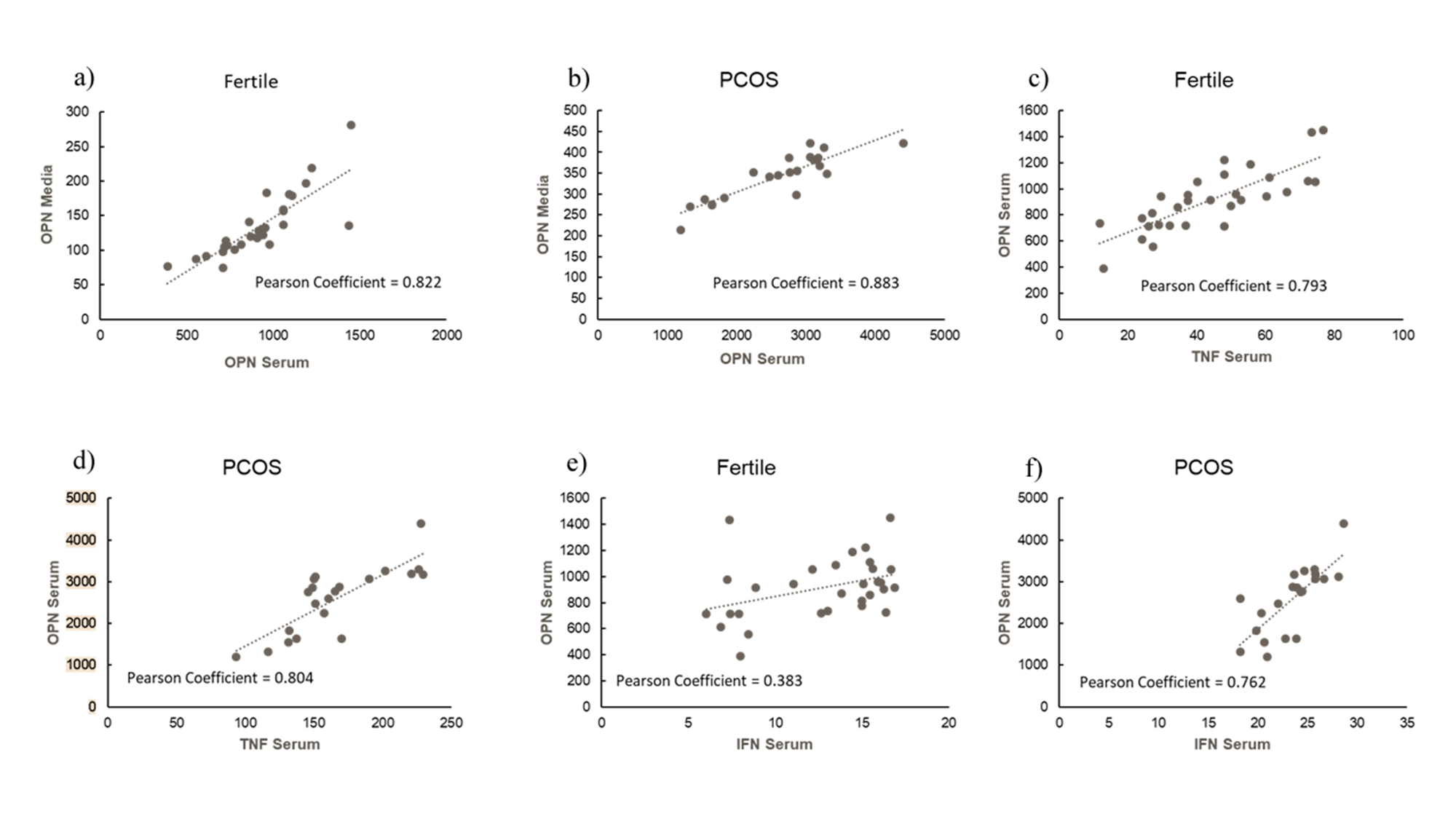

Supplement: Supplementary file 3 — . Significant positive associations between OPN levels in serum vs OPN media levels (Panel A & B), serum TNF levels (Panel C & D) and serum IFNγ levels (Panel E & F) in fertile and PCOS patients. Values are expressed as the average ± SD. Statistical analysis of the data was performed using a Student t test and significance differences described as *, P ≤ 0.05, **, P ≤ 0.01 and ***, P ≤ 0.001. Pearson coefficient = r. (PNG 6596 kb) [file 109_2020_1985_Fig8_ESM.png]

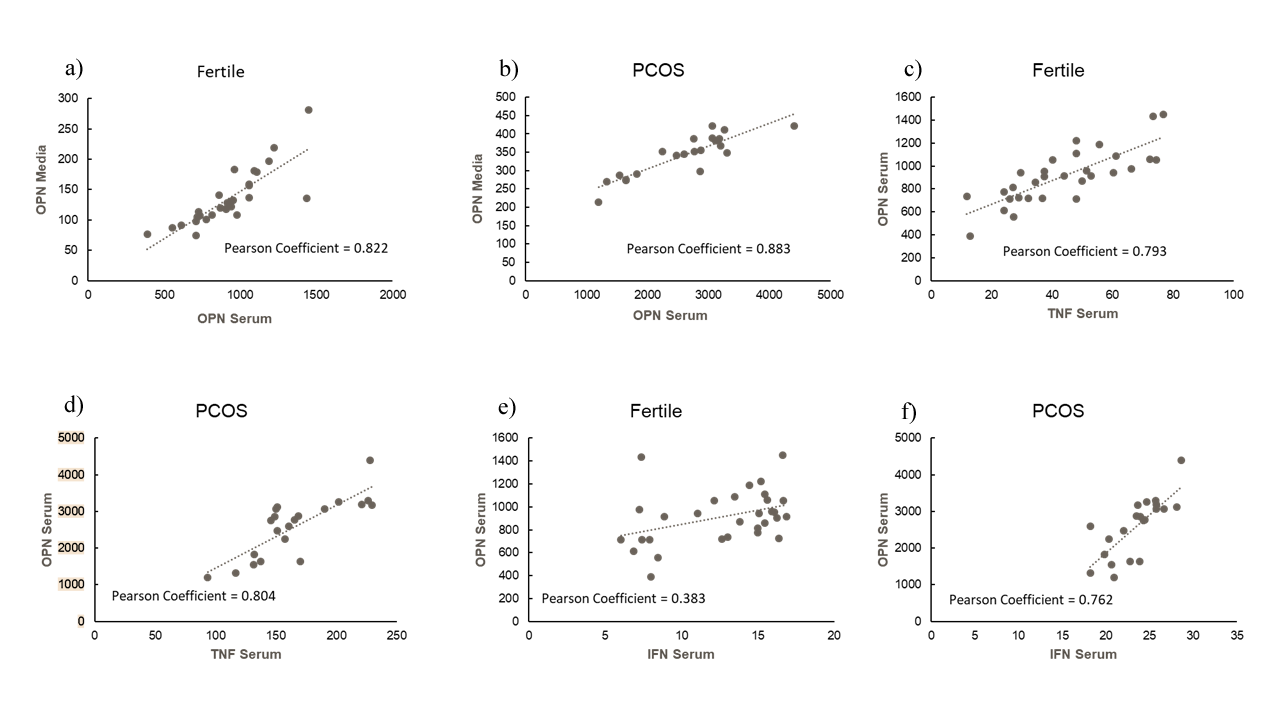

Supplement: Supplementary file 4 — High Resolution Image (TIF 2703 kb) [file 109_2020_1985_MOESM2_ESM.tif]

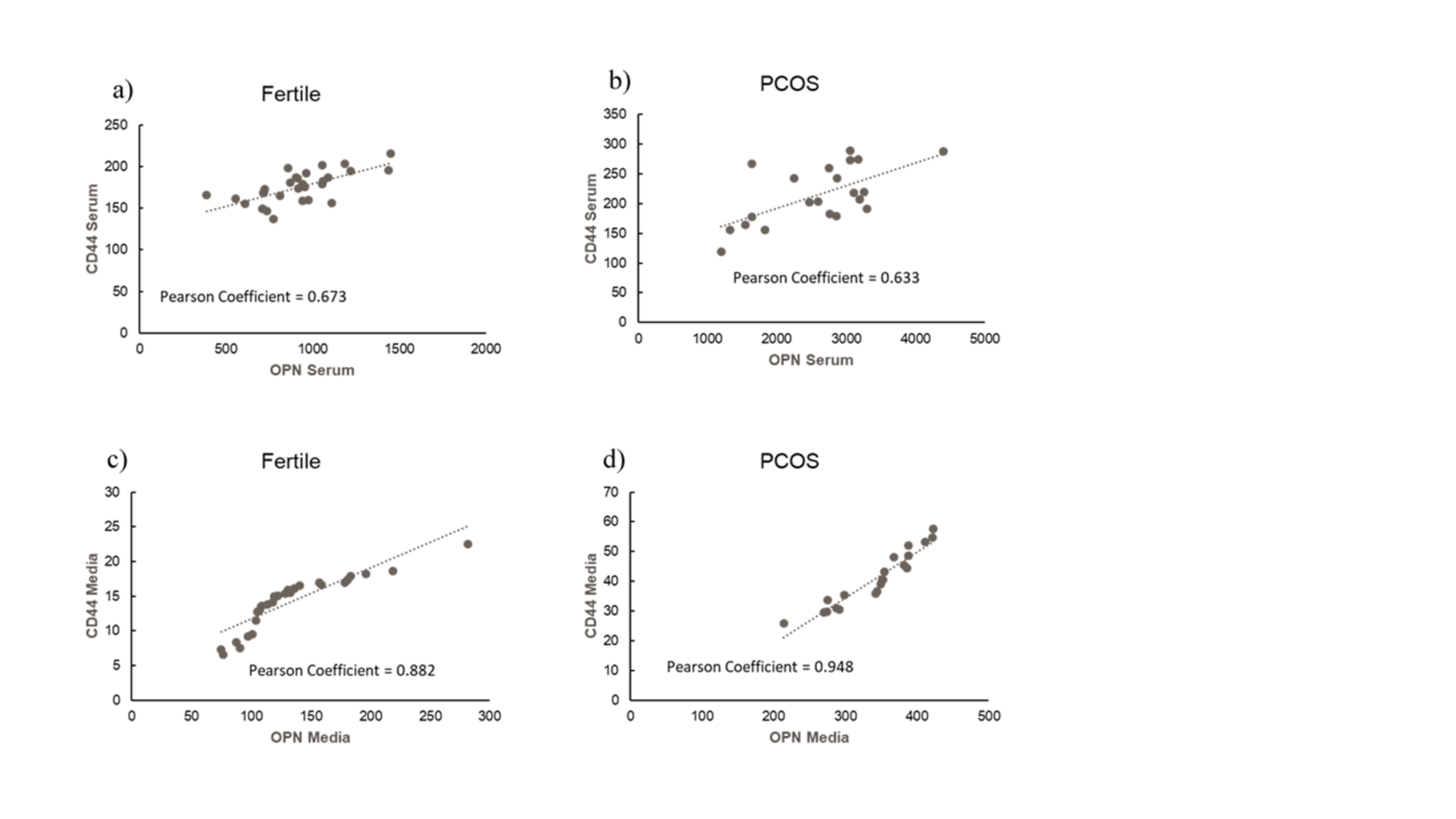

Supplement: Supplementary file 5 — . Positive correlations between serum CD44 and OPN levels in fertiles (A) and PCOS groups (B). Positive correlations between CD44 vs OPN levels in media from fertile (C) and PCOS (D) patients. Values are expressed as the average ± SD. Statistical analysis of the data was performed using a Student t test and significance differences described as *, P ≤ 0.05, **, P ≤ 0.01 and ***, P ≤ 0.001. Pearson coefficient = r. (PNG 6596 kb) [file 109_2020_1985_Fig9_ESM.png]

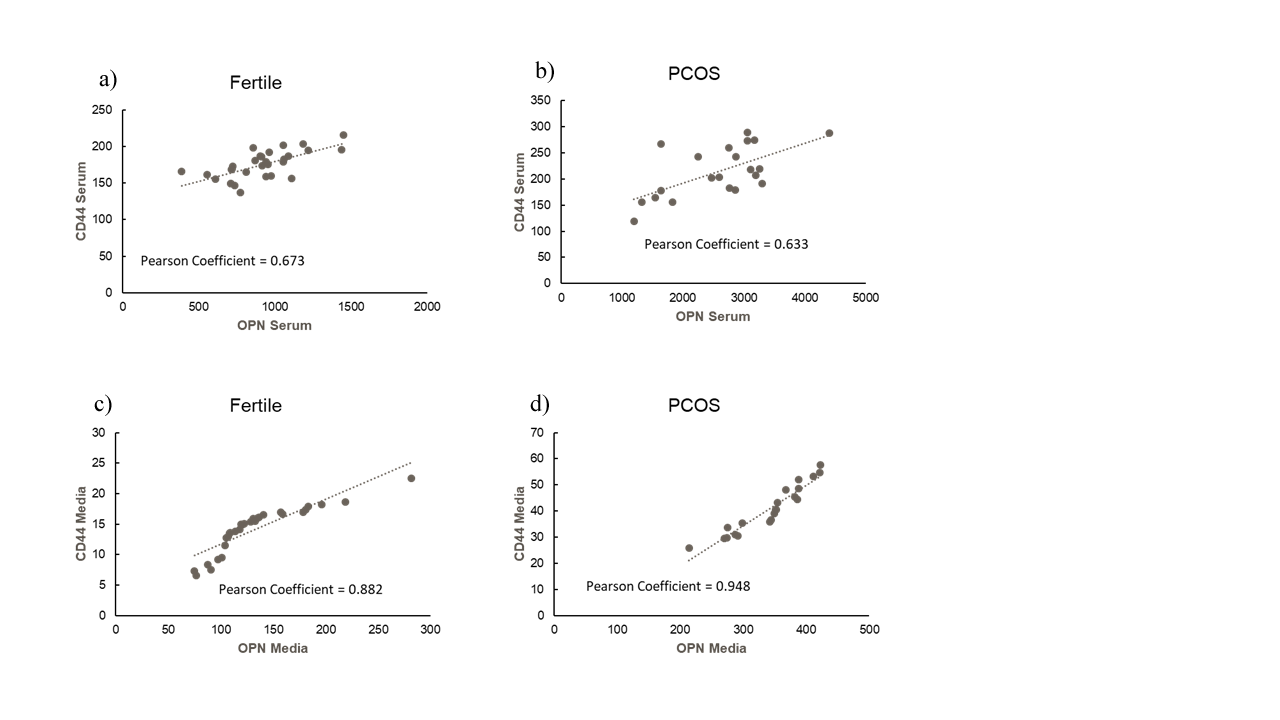

Supplement: Supplementary file 6 — High Resolution Image (TIF 2702 kb) [file 109_2020_1985_MOESM3_ESM.tif]
